# Changes in Anthropometric and Performance Parameters in High-Level Endurance Athletes during a Sports Season

**DOI:** 10.3390/ijerph18052782

**Published:** 2021-03-09

**Authors:** Javier Alves, Gema Barrientos, Víctor Toro, Esther Sánchez, Diego Muñoz, Marcos Maynar

**Affiliations:** 1 Department of Sport Science, Faculty of Education, Pontifical University of Salamanca, 37007 Salamanca, Spain; fjalvesva@upsa.es (J.A.); esanchezmo@upsa.es (E.S.); 2 Department of Physiology, Faculty of Sports Science, University of Extremadura, 10003 Cáceres, Spain; vtororom@alumnos.unex.es (V.T.); diegomun@unex.es (D.M.); mmaynar@unex.es (M.M.)

**Keywords:** skinfolds, fat mass, muscle mass, maximal oxygen consumption, endurance

## Abstract

Several anthropometric and performance parameters related to aerobic metabolism are associated with performance in endurance runners and are modified according to the training performed. The objective of this study was to investigate the ergospirometric and body composition changes in endurance runners during a sports season in relation to their training. Twenty highly trained men endurance runners performed an incremental test until exhaustion (initial, and at 3, 6, and 9 months) on a treadmill to determine maximal oxygen consumption (VO_2_ max), second ventilatory threshold (VT_2_), and their associated running speeds. Skinfolds, perimeters, and weights were measured. No changes were obtained in VO_2_ max or VT_2_ during the study, although their associated running speeds increased (*p* < 0.05) after 3 months of the study. Decreases in fat mass (*p* < 0.05) and muscle mass (*p* < 0.05) were observed at the end of the season (9 months). Changes occurred in the different skinfolds according to the characteristics of the training performed during the season. In conclusion, vVO_2_ max and vVT_2_ increase with a greater volume of kilometres trained and can be adversely affected by loss of muscle mass.

## 1. Introduction

Different physiological and anthropometric parameters have been investigated for being associated with performance in endurance runners [1,2]. Parameters related to aerobic function such as maximal oxygen consumption (VO_2_ max) and the second ventilatory threshold (VT_2_) or anaerobic threshold (AT) have been identified as determining factors in endurance runners [3]. Performing an incremental stress test to voluntary exhaustion with gas exchange measurement is the most commonly used method to determine these parameters in endurance runners [4].

Highly trained elite endurance runners present high values of VO_2_ max [5], although it is not a determining parameter for performance prediction in homogeneous groups [6]. Previous studies reported no difference in VO_2_ max values between middle- and long-distance (3000–10000 m) well-trained runners [7,8]. Running speed at maximal oxygen consumption (vVO_2_ max) has been established as a better performance predictor, since highly trained endurance runners can maintain this speed from four to seven minutes, although there is variability among subjects [9].

Another performance factor is VT_2_, which shows the ability to sustain the highest percentage of VO_2_ max for a long time [10]. Research in high level endurance runners has reported higher values of VT_2_ and its associated velocity (vVT_2_) than in recreational runners; therefore, high-level runners can maintain faster running speeds for longer [11,12].

Likewise, endurance runners must have adequate anthropometric and body composition parameters, since an excess of weight without an increase in strength will cause a decrease in running speed [8]. Several anthropometric and body composition values are associated with running performance, such as body weight, fat mass, muscle mass, calf skinfold, and sum of 6 (∑6) skinfolds [1,7,13,14].

An appropriate energy intake in runners is crucial to preserve optimal body function and maintain a lean body composition to enhance performance [15]. The use of questionnaires that collect data on the foods and beverages consumed by the subjects over several days to determine the nutritional intake is common in athletes [16].

Periodisation of the different training loads that runners perform, changes in volume and intensity during the sports season, influence the adaptations and performance in the runners [17]. There are few longitudinal studies in homogeneous groups of runners that include different evaluation moments and have investigated the changes in anthropometrics and performance parameters throughout a sports season as a consequence of the training sessions performed [18].

Therefore, the main objective of this study was to measure the changes in body composition, anthropometric, and performance parameters related to aerobic function by measuring skinfold thickness and performing an incremental test until exhaustion in highly trained endurance men runners at four evaluation points (each 3 months) throughout a sports season in relation to the volume and intensity of km trained per week.

## 2. Materials and Methods

### 2.1. Participants

Twenty highly trained men endurance runners (23 ± 3 years old; height: 1.77 ± 0.05 m) participated in the research and were studied at four time points during the athletic season, in the first week of October, January, April, and July.

All subjects participated voluntarily, were informed about the purpose of the study, and gave their written consent. The study was approved by the Ethics Committee of the University of Extremadura (Register number 52/2012), and all procedures were in accordance with the Helsinki Declaration ethical guidelines updated at the World Medical Assembly in Fortaleza (Brazil) in 2013 for investigations with human subjects.

Athletes were recruited from different training groups from the same region. They competed in cross country, road races, and 1500 to 5000 m race modalities, and they had a personal best of 3:37.79–4:08.24 for 1500 m and 13:11.01 and 15:10.35 in 5000 m. The inclusion criteria were to have been training regularly for at least 5 years, performing at least 6 sessions and 70 km per week (km/w) during the season, and to have competed in regional, national, and international events. Exclusion criteria were not having trained for extended periods due to injury or any other reason, or having changed nutritional habits and diet.

Before the study, the runners were informed about the energy and macronutrient intake guidelines for athletes established by the American College of Sports Medicine [19] that they had to follow according to the training carried out and their personal characteristics.

### 2.2. Training Characteristics

**Figure 1:**
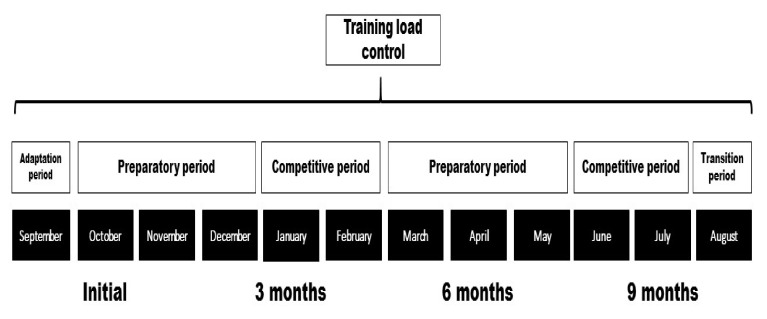
Periodisation during the season.

All the runners performed a traditional periodisation with two competitive periods (Figure 1). The first competitive period for the runners began in January through February, when runners competed in cross-country competitions, and the second one was in June and July, when runners competed in track and field events of between 1500 and 5000 m. The first preparatory period began in October through December, and the second one was from March to May. In the preparatory periods, runners trained high volumes of km/w at low and moderate intensities. In competitive periods, the runners reduced the volume of km/w but trained at high intensities. Before the initial measurement, the athletes had completed four weeks of adaptive training after the rest period of the previous season. The characteristics of the weekly and accumulated training during the season are detailed in Table 1. A pulsometer equipped with GPS was used to track the training loads during the season. Runners used their usual equipment (Vantage M, Polar, Finland; 5, Suunto, Finland; Forerunner 235, Garmin, USA). Runners used the clocks daily in their workouts, and weekly intensity and volume data were collected in the tests.

Depending on the period of the season, they also performed two weekly sessions of resistance training with a high volume (3–5 sets of 8–25 repetitions of whole-body exercises) and moderate intensity (30–70% of 1 Repetition maximum and plyometric training.

### 2.3. Nutritional Assessment

The macronutrient composition of the participants’ diets was determined using a database [20]. The athletes completed a 3-day nutritional questionnaire on two working days and one weekend day, where they indicated the amount (in grams) of all food ingested on those days.

### 2.4. Anthropometric Measures

The participants’ characteristics were measured in the morning and always at the same time (9:00–10:00). Participants were informed that they should go to the laboratory well hydrated after an overnight fast and refrain from intense training or competition for at least 72 h prior to testing.

Body weight was measured to the nearest of 0.01 kg using a calibrated electronic digital scale (Seca 769, Hamburg, Germany). Height was measured with an accuracy of 0.1 cm using a wall mounted stadiometer (Seca 220, Hamburg, Germany). Arm and leg perimeters were obtained (in a relaxed 90º position) with an accuracy of ± 1 mm using a tape (Seca 212. Hamburg, Germany). Skinfold thicknesses (abdominal, suprailiac, tricipital, subscapular, thigh, and leg) were measured with a Harpenden calliper (Holtain skinfold calliper, Crosswell, UK). Measurements were taken three times by an expert in kinanthropometry techniques (accredited level 1) who had previously shown a test–retest reliability of r > 0.9, in accordance with the recommendations of the International Society for the Advancement of Kinanthropometry [21]. Body composition was calculated according to the indications of the Spanish Kinanthropometry group [22].

### 2.5. Physical Performance Evaluation

After taking anthropometric measurements, to measure the runners’ ergospirometric parameters and performance, they carried out an incremental test until exhaustion on a treadmill (Powerjog, Birmingham, UK) with an ergospirometer system equipped with a gas analyser (Metamax, Cortex Biophysik, Germany). A pulsometer (Vantage M, Polar, Finland) was used to evaluate the maximal heart rate.

After a 10 min warm-up, the runners initiated the test at a speed of 10 km/h, which increased by 1 km/h every 400 m until voluntary exhaustion. VO_2_ max was determined according to the following criteria: there had to be a plateau in oxygen uptake (VO_2_), an increment in carbon dioxide (CO_2_) elimination, and an increment in the ventilatory volume (VE) induced by the increases in the test velocity and the respiratory exchange ratio (RER) had to exceed 1 [23]. The aerobic threshold (VT_1_) and VT_2_ were determined according to the three-phase model to monitor training [24].

### 2.6. Statistical Analysis

Statistical analysis was carried out with IBM SPSS Statistical software version 21.0 (IBM Co., Armonk, NY, USA). The results are expressed as x ± sd, where x is the mean value and sd is the standard deviation. Before the analyses, all variables were checked for normality of distribution with Kolmogorov–Smirnov tests. The data were analysed by repeated measurements analysis of variance (ANOVA) and with the Bonferroni post hoc test for moment/period as the categorical variable. Partial eta squared (ηp^2^) was used as an effect size measure of ANOVA. Threshold values for assessing magnitudes of standardised effects were ηp^2^ ≥ 0.01, ηp^2^ ≥ 0.06, and ηp^2^ ≥ 0.14 for small, medium, and large, respectively [25]. The equality of variances between the differences was assessed with Mauchly’s test of sphericity. When sphericity was violated, Greenhouse–Geisser corrected p-values were used. A simple linear regression model was used to determine associations between ergospirometric and body composition parameters. Pearson’s correlation coefficient (r), the β coefficients, and determination coefficients (R^2^) were calculated. A *p* ≤ 0.05 was considered statistically significant.

## 3. Results

Nutritional intake of energy and macronutrients in the runners during the season is shown in Table 2. There were no significant differences in energy and macronutrient intake during the season.

Table 3 shows the changes in the ergospirometric parameters, running speeds associated with VO_2_ max and VT_2_, as well as the performance results obtained in the different incremental tests of the runners during the sports season.

Table 4 shows the different weights, skinfolds and perimeters of the runners at the four time points during the sports season.

In this study, there were intra-group differences in body mass, fat mass, abdominal skinfolds, and calf skinfolds (*p* < 0.05).

At 3 months, a significant increase (*p* < 0.01) in vVT_2_ and vVO_2_ max was observed (*p* < 0.01). In relation to the skinfolds, a decrease in the calf skinfold was observed (*p* < 0.01).

At 6 months, an increase in vVO_2_ max was observed (*p* < 0.01), together with a decrease in fat mass (*p* < 0.05) and in the abdominal skinfold (*p* < 0.01) compared to the beginning of the season. In addition, a decrease in body mass (*p* < 0.05) and the abdominal skinfold were reported, which was accompanied by an increase in the calf skinfold (*p* < 0.05) compared to 3 months of training.

At 9 months, a decrease in fat and muscle mass was reported (*p* < 0.05) accompanied by a decrease in the abdominal skinfold (*p* < 0.05) compared to the beginning of the season. An increase in the calf skinfold (*p* < 0.05) and decreases in the abdominal skinfold and body mass were also reported with respect to 3 months of training.

Table 5 shows the main correlations found between performance parameters and anthropometric values related to aerobic function and their associated speeds. In our runners, a very significant negative relationship was observed between VO_2_ max and fat mass (r= −0.405; β: −2.831; *p* < 0.001) and ∑6 skinfolds (r= −0.429; β: −0.353; *p* < 0.001). An inverse relationship between vVO_2_ max with fat mass (r= −0.291; β: −0.361; *p* = 0.009) and ∑6 skinfolds (r= −0.424; β: −0.062; *p* < 0.001) was also reported. 

**Figure 2:**
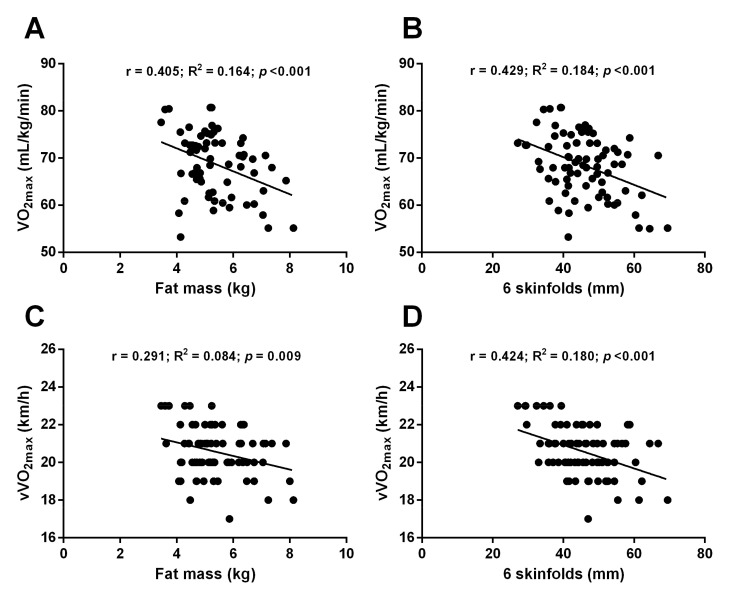
Linear regressions of significant correlations between performance parameters and anthropometric values. (**A**): linear regression between fat mass and VO_2max_; (**B**): linear regression between ∑6 skinfolds and VO_2max_; (**C**): linear regression between fat mass and vVO_2max_; (**D**): linear regression between ∑6 skinfolds and vVO_2max_; r: Pearson’s coefficient of correlation; R^2^: coefficient of determination; *p*: *p*-value; VO_2_ max: maximal oxygen consumption; vVO_2_ max: running speed at maximal oxygen consumption.

Figure 2 shows linear regressions of significant correlations between performance parameters and anthropometric values.

## 4. Discussion

Our longitudinal study was designed to observe the changes in the anthropometric and performance parameters during a sports season in highly trained endurance runners in relation to the training performed.

In the study, runners had high VO_2_ max values without significant changes during the season. High VO_2_ max is required for endurance runners [26], and our runners had similar VO_2_ max values to those recorded in other studies [11,18]. In addition, it has been widely reported that VO_2_ max does not change significantly with training in highly trained athletes [27]. Jones [28] found no changes in this parameter over a 5-year study period in a well-trained runner.

Adequate anthropometric and body composition parameters are associated with performance in runners [29]. Estimating body composition parameters by measuring different skinfolds is a widely used method in runners [30], and it is a valid and reliable technique when it is performed by a certified and trained operator [31]. In this study, low values in ∑6 skinfolds of our runners were observed, although they were higher than those reported in studies with elite runners [30,32]. There were no significant changes of ∑6 skinfolds during the season. In elite athletes, it is necessary to periodise body composition during the season, as maintaining extremely low values of fat mass for extended periods could be negative for their health [33].

For a better understanding, we will discuss the changes in performance parameters, body composition, and different skinfolds by periods, to be able to relate them to the training performed.

After 3 months, a significant improvement in vVO_2_ max and vVT_2_ and a decrease in the calf skinfold was observed in the runners. In this period, the runners performed the highest volume km/w during the season. In a previous study, Jones [28] reported a decrease in VO_2_ max in an elite athlete over a 5-year period, with performance improvements attributed to an increase in vVT_2_. In another study, Nicholson et al. [34] showed that there is a strong correlation between vVT_2_ and performance in endurance runners. It seems to be a very sensitive parameter to improvement with optimal training [11]. In addition, it has been documented that fat mass loss is specific to the muscle groups most used during activities performed in training [7]. Arrese et al. [1] obtained a correlation between the calf skinfold and performance in high-level middle- and long-distance runners. It is known that a lower weight in the distal part of the legs would require less work in their movement during the race [35], improving performance as a consequence of a lower energy expenditure [36]. A 10% increase in aerobic demand has been reported for each kilo of extra weight carried on the distal part of the legs; however, when the weight is carried on the trunk, it only increases by 1% [8]. In addition, Tjelta et al. [12] reported a lower energy expenditure (running economy) in athletes who trained a greater volume of km per week. The increase in vVO_2_ max and the vVT_2_ of the runners in our study could be explained by a lower energy demand, due to the decrease in the calf skinfold that reduced the mechanical work of the runners at the same running intensities. 

At 6 months, a decrease in body mass, fat mass, and the abdominal skinfold with respect to the initial evaluation and an increase in the calf skinfold in relation to 3 months were reported. In this period, after the end of the first competitive period, the runners performed less volume and increased intensity, with more km/w trained at high intensity (>VT_2_). Several studies have concluded that high-intensity interval training (HIIT) appears to be a method that causes a greater loss of fat mass [37] from the trunk and abdominal skinfold compared to endurance training [38,39], when participants are physically and motivationally prepared to perform for a certain time and at an adequate intensity [40]. In addition, Davies et al. [41] reported that the abdomen fat mass acts as the primary region for the change in fat tissue in sportsmen during a season. In this period, an increase in vVO_2_ max was observed related to the initial measurement. It has been widely documented that HIIT sessions are a very effective method for improving performance in endurance runners [42,43]. Ingham et al. [44] reported improvements in vVo_2_ max in an elite runner that prioritised training sessions with intensities above VT_2_ and VO_2_ max with recovery sessions with low intensities (VT_1_). In this vein, runners’ adaptations occur mainly at the intensities that are most stimulated during training sessions [4].

At 9 months, the runners reported a decrease in fat mass with respect to the initial measurement and in the abdominal skinfold compared to 3 months. During this period, runners periodise their training with the aim of optimising their fitness to compete in track and field events of less distance (1500 to 5000 m) and shorter duration at a higher speed [42]. In addition, the runners had accumulated 6 months of training, and they did not perform such a high volume of aerobic training. In competitive periods, runners perform more high-intensity weekly sessions, a lower total volume of km/week, but with the highest number of km/week performed above VT_2_ and VO_2_ max [45]. The results obtained are in relation to the aforementioned, as performing high-intensity interval training reduces the abdominal skinfold in runners [37,38,39].

We also reported a decrease in the calf skinfold and fat mass in the runners compared to the initial measurement, which are variables associated with successful runners in endurance events [1], although in this period, there was no improvement in vVT_2_ or vVO_2_ max. In addition, in this period, a decrease in muscle mass was found in our runners compared to the initial value. It has been documented that high-intensity training sessions increase muscle damage, which without proper recovery can induce muscle catabolism [46]. In a recent study, it has been reported that endurance athletes have difficulty maintaining optimal nutrition, which affects anabolic hormones such as testosterone as well as performance toward the end of the season [47].

In addition, several authors have indicated that strength improvements increase performance in middle and long-distance runners [48,49]. A decrease in muscle mass leads to a loss of muscle strength [50], and consequently, vVO_2_ max and running speed would be negatively affected [48]. 

Another parameter such as fat mass decreased at 6 and 9 months with respect to the initial evaluation. As previously mentioned, endurance athletes have difficulty maintaining adequate energy availability during the season, causing decreases in fat mass [47].

There was also a decrease in body weight at 6 and 9 months compared to 3 months as a consequence of decreases in fat and muscle mass.

No significant changes in time and total metres were reported in the maximal incremental test until exhaustion during the season.

Regarding the correlation study, an inverse relation was observed between fat mass and ∑6 skinfolds with VO_2_ max and vVO_2_ max. It has been widely reported that high values of body fat and skinfolds have a negative effect on performance in endurance runners [1,8,29].

The limitations of the study were the impossibility of using Dual-energy X-ray absorptiometry (DXA) or Bioelectrical Impedance Analysis (BIA) to measure changes in body composition that would have reported more accurate data. Running economy could not be measured due to the protocol used, although the improvements in this parameter are related to the greater experience and the training km of the runners [12]. Another limitation was that the runners’ diets could not be monitored throughout the season; it was only possible to monitor the week prior to the evaluations to verify that there were no changes in diet.

## 5. Conclusions

Endurance runners who train high weekly volumes with long-duration sessions at low-moderate intensity favour the decrease in the calf skinfold, which is positively related to performance in endurance running. Training periods with a lower weekly volume but more km/week performed at high intensity favour a decrease in the abdominal skinfold.

Performance-related parameters in endurance runners such as vVO_2_ max and vVT_2_ improved with specific training sessions at those intensities.

However, endurance runners must control training load, as excessively high-intensity training can lead to muscle catabolism, which would negatively affect strength.

Based on these results, high-level endurance runners should perform high-intensity training sessions (>VT_2_) to improve performance. Periodising high volumes of km /w is necessary to have a lower energy expenditure in vVT_2_ and vVO_2_ max. It is of special interest to control the high-intensity interval training sessions and load in runners after long periods of training and competition, where there is greater accumulated fatigue, making it necessary to anticipate recoveries and nutritional strategies.

## Figures and Tables

**Table 1:** Training loads in the runners during the season.

Training Load	Initial	3 Months	6 Months	9 Months
Total (km/week)	85.12 ± 13.1	106.52 ± 15.78	93.53 ± 14.56	74.55 ± 13.8
>VT_2_ (km/week)	4.10 ± 0.5	12.68 ± 2.03	18.66 ± 2.8	16.45 ± 3.1
≤VT_2_ (km/week)	81.02 ± 12.6	93.84 ± 13.75	74.87 ±11.4	58.10 ± 10.7

≤VT_2_ intensity below second ventilatory threshold; >VT_2_ intensity above second ventilatory threshold.

**Table 2:** Energy and macronutrient intake in the runners during the season.

Parameters	Initial	3 Months	6 Months	9 Months	ηp^2^
Energy (kcal/d)	2855 ± 511.3	2795.4 ± 427.2	2902.4 ± 522.5	3108.7 ± 770.2	0.07
CH (g/kg/d)	5.26 ± 1.21	5.28 ± 1.14	6.25 ± 1.38	6.13 ± 1.50	0.03
Protein (g/kg/d)	1.73 ± 0.79	1.69 ± 0.35	1.85 ± 0.53	1.89 ± 0.63	0.05
Lipids (g/kg/d)	1.78 ± 0.40	1.63 ± 0.28	1.58 ± 0.52	1.72 ± 0.74	0.05

CH: carbohydrates.

**Table 3:** Ergospirometric and performance parameters of the runners.

Parameters	Initial (M ± SD)	3 Months (M ± SD)	6 Months (M ± SD)	9 Months (M ± SD)	*p*	F	ηp^2^
	(CI 95%) SEM	(CI 95%) SEM	(CI 95%) SEM	(CI 95%) SEM			
VO_2_ max (mL/kg/min)	68.02 ± 4.73 (65.80–70.23) 1.05	67.72 ± 9.76 (65.14–71.83) 1.59	68.65 ± 7.14 (65.66–72.06) 1.53	68.80 ± 7.50 (65.29–72.31) 1.67	0.306	1.269	0.07
VT_2_ (% VO_2_ max)	91.02 ± 2.43 (89.86–92.19) 0.55	91.49 ± 3.59 (91.02–94.48) 0.82	91.34 ± 3.08 (89.85–92.46) 0.62	90.96 ± 2.07 (89.27–92.05) 0.42	0.675	0.511	0.04
vVO_2_ max (Km/h)	20.20 ± 0.98 (19.68–20.61) 0.22	20.90 ± 1.13 ** (20.24–21.33) 0.26	20.71 ± 1.22 ** (20.15–21.27) 0.26	20.67 ± 1.75 (19.82–21.47) 0.39	0.095	2.071	0.12
vVT_2_ (Km/h)	19.27 ± 0.80 (18.45–20.06) 0.19	20.00 ± 0.89 ** (19.19–20.36) 0.16	19.65 ± 1.27 (18.81–19.89) 0.21	19.41 ± 1.65 (18.14–20.33) 0.28	0.182	1.889	0.08
RER	1.05 ± 0.03 (1.03–1.07) 0.00	1.05 ± 0.05 (1.03–1.07) 0.00	1.05 ± 0.04 (1.02–1.07) 0.01	1.04 ± 0.04 (1.03–1.06) 0.01	0.877	0.088	0.02
Maximum heart rate	190.6 ± 9.45 (185.4–193.7) 1.97	192.9 ± 8.01 (190.9–198.3) 1.77	193.3 ± 8.95 (189.1–196.8) 1.85	193.8 ± 7.52 (189.1–197.3) 1.95	0.786	0.485	0.04
Distance (m)	4543.3 ±42.1 (4346–4740) 94.16	4608.6 ± 49.8 (4338–4800) 109.9	4677.0 ± 56.2 (4450–4970) 124.77	4720.7 ± 65.9 (4416–5025) 145.54	0.423	0.790	0.06
Time (min)	20.30 ± 1.85 (19.45–21.18) 0.41	21.00 ± 2.17 (19.98–22.13) 0.51	21.10 ± 2.04 (20.17–21.99) 0.43	20.70 ± 2.67 (19.47–21.97) 0.59	0.709	0.141	0.03

RER: respiratory exchange ratio; VO_2_ max: maximal oxygen consumption; VT_2_: second ventilatory threshold; vVO_2_ max: running speed at maximal oxygen consumption; vVT_2_: running speed at second ventilatory threshold; ** *p* < 0.01 difference between 0 vs. 3–6; SEM: standard error of the mean; CI: confidence interval; *p*: value intergroup.

**Table 4:** Body composition parameters of the runners during the season.

Parameters	Initial (M ± SD)	3 Months (M ± SD)	6 Months (M ± SD)	9 Months (M ± SD)	p	F	ηp^2^
	(CI 95%) SEM	(CI 95%) SEM	(CI 95%) SEM	(CI 95%) SEM			
Body mass (kg)	65.35 ± 7.46 (61.85–68.83) 1.66	65.31 ± 7.53 (61.26–68.42) 1.70	64.58 ± 7.21 ^#^ (61.69–68.37) 1.60	64.59 ± 7.47 ^#^ (61.09–68.08) 1.67	0.030	2.557	0.21
Bone mass (kg)	11.96 ± 1.02 (11.49–12.44) 0.22	11.84 ± 1.09 (11.27–12.34) 0.25	11.96 ± 1.13 (11.47–12.48) 0.24	11.98 ± 1.09 (11.4612.48) 0.24	0.950	0.096	0.02
Fat mass (kg)	5.59 ± 1.27 (4.99–6.17) 0.28	5.41 ± 1.10 (4.83–6.10) 0.25	5.25 ± 0.86 * (4.89–5.68) 0.18	5.22 ± 0.99 * (4.87–5.77) 0.22	0.012	2.737	0.29
Muscle mass (kg)	32.12 ± 4.09 (30.20–34.03) 0.91	32.26 ± 4.09 (30.04–33.90) 0.91	31.71 ± 4.00 (30.12–33.85) 0.89	31.72 ± 4.16 * (29.77–33.26) 0.93	0.072	2.433	0.15
Abdominal S. (mm)	9.70 ± 2.65 (8.46–10.93) 0.59	9.87 ± 2.82 (8.45–11.24) 0.66	8.22 ± 1.86 ** (7.45–9.16) 0.40	8.84 ± 2.34 ^#^ (7.73–9.93) 0.52	0.021	2.776	0.26
Suprailiac S. (mm)	5.68 ± 1.27 (5.08–6.27) 0.28	6.14 ± 0.88 (5.67–6.52) 0.20	5.51 ± 0.98 (5.05–6.08) 0.21	5.99 ± 1.17 (5.44–6.53) 0.26	0.276	1.749	0.08
Subscapular S. (mm)	8.23 ± 1.53 (7.51–8.94) 0.34	8.40 ± 1.94 (7.45–9.37) 0.45	7.87 ± 1.39 (7.26–8.50) 0.29	8.20 ± 1.66 (7.41–8.97) 0.37	0.785	0.355	0.04
Tricipital S. (mm)	6.24 ± 1.58 (5.50–6.97) 0.35	6.06 ± 1.34 (5.36–6.68) 0.31	5.98 ± 1.35 (5.40–6.61) 0.28	6.52 ± 1.72 (5.70–7.32) 0.38	0.689	0.292	0.03
Front Thigh S. (mm)	8.48 ± 3.12 (7.01–9.93) 0.69	8.76 ± 2.88 (7.30–10.14) 0.67	8.67 ± 2.64 (7.52–9.87) 0.56	8.18 ± 2.30 (7.10–9.25) 0.51	0.717	0.169	0.03
Calf S. (mm)	8.26 ± 3.15 (6.77–9.54) 0.65	6.70 ± 2.09 ** (5.59–7.62) 0.48	8.65 ± 2.44 ^##^ (7.54–9.53) 0.47	7.74 ± 2.15 * ^##^ (6.73–8.74) 0.48	0.023	2.617	0.26
∑ 6 skinfolds (mm)	46.59 ±11.11 (41.35–51.61) 2.44	45.92 ± 8.80 (41.30–50.14) 2.10	44.89 ±7.60 (41.62–48.41) 1.62	45.46 ± 8.54 (41.46–49.44) 1.90	0.764	0.293	0.04
Arm P. (cm)	27.61 ±2.37 (26.49–28.71) 0.53	27.54 ± 2.49 (26.30–28.76) 0.58	27.11 ±2.39 (26.07–28.19) 0.50	27.09 ± 2.67 (25.83–28.33) 0.59	0.513	0.433	0.05
Leg P. (cm)	36.20 ± 2.01 (35.25–37.14) 0.44	36.25 ± 1.88 (35.22–36.96) 0.41	36.14 ±1.76 (35.44–37.11) 0.40	36.03 ± 1.99 (35.09–36.95) 0.44	0.575	0.431	0.04

S: skinfold; P: perimeter; ∑ 6 skinfolds: sum six skinfolds. * *p* < 0.05 difference between 0 vs. 3–6–9; ** *p* < 0.01 difference between 0 vs. 3–6–9; ^#^ *p* < 0.05 difference between 3 vs. 6–9; ^##^ *p* < 0.01 difference between 3 vs. 6–9; SEM: standard error of the mean; CI: confidence interval; p: value intergroup.

**Table 5:** Simple linear regressions between performance parameters and anthropometric values.

Parameters	VO_2_ max (mL/kg/min)				vVO_2_ max (km/h)			
	r	R^2^	β	*p*	r	R^2^	β	*p*
Fat mass (kg)	−0.405	0.164	−2.831	<0.001	−0.291	0.084	−0.361	0.009
Muscle mass (kg)	−0.195	0.038	−0.358	0.083	0.016	0.000	0.005	0.888
Bone mass (kg)	−0.168	0.028	−1.033	0.135	0.039	0.002	0.049	0.728
∑ 6 skinfolds (mm)	−0.429	0.184	−0.353	<0.001	−0.424	0.180	−0.062	<0.001
	VT_2_ (% VO_2_ max)				vVT_2_ (km/h)			
	r	R^2^	β	*p*	r	R^2^	β	*p*
Fat mass (kg)	0.035	0.001	0.092	0.760	−0.034	0.001	−0.036	0.763
Muscle mass (kg)	0.001	0.000	0.000	0.996	0.122	0.015	0.033	0.282
Bone mass (kg)	0.044	0.002	0.115	0.700	0.096	0.009	0.092	0.396
∑ 6 skinfolds (mm)	−0.050	0.002	−0.016	0.661	−0.056	0.003	−0.007	0.620

r: Pearson’s coefficient of correlation; β: beta coefficient; R^2^: coefficient of determination; *p*: *p*-value; VO_2_ max: maximal oxygen consumption; vVO_2_ max: running speed at maximal oxygen consumption; VT_2_: second ventilatory threshold.
